# Application of Rhizobacteria, *Paraburkholderia fungorum* and *Delftia* sp. Confer Cadmium Tolerance in Rapeseed (*Brassica campestris*) through Modulating Antioxidant Defense and Glyoxalase Systems

**DOI:** 10.3390/plants11202738

**Published:** 2022-10-16

**Authors:** Md. Rakib Hossain Raihan, Mira Rahman, Nur Uddin Mahmud, Malay Kumar Adak, Tofazzal Islam, Masayuki Fujita, Mirza Hasanuzzaman

**Affiliations:** 1 Department of Agronomy, Sher-e-Bangla Agricultural University, Dhaka 1207, Bangladesh; 2 Institute of Biotechnology and Genetic Engineering (IBGE), Bangabandhu Sheikh Mujibur Rahman Agricultural University, Gazipur 1706, Bangladesh; 3 Department of Botany, University of Kalyani, Nadia 741235, West Bengal, India; 4 Laboratory of Plant Stress Responses, Faculty of Agriculture, Kagawa University, Miki-cho, Kita-gun, Takamatsu 761-0795, Japan

**Keywords:** abiotic stress, methylglyoxal, oilseed crop, plant-microbe interaction, ROS, soil heavy metals

## Abstract

We investigated the role of two different plant growth-promoting probiotic bacteria in conferring cadmium (Cd) tolerance in rapeseed (*Brassica campestris* cv. BARI Sarisha-14) through improving reactive oxygen species scavenging, antioxidant defense, and glyoxalase system. Soil, as well as seeds of rapeseed, were separately treated with probiotic bacteria, *Paraburkholderia fungorum* BRRh-4 and *Delftia* sp. BTL-M2. Fourteen-day-old seedlings were exposed to 0.25 and 0.5 mM CdCl_2_ for two weeks. Cadmium-treated plants resulted in a higher accumulation of hydrogen peroxide, increased lipid peroxidation, electrolyte leakage, chlorophyll damage, and impaired antioxidant defense and glyoxalase systems. Consequently, it reduced plant growth and biomass production, and yield parameters. However, probiotic bacteria-inoculated plants significantly ameliorated the Cd toxicity by enhancing the activities of antioxidant enzymes (ascorbate peroxidase, dehydroascorbate reductase, monodehydroascorbate reductase, glutathione reductase, glutathione peroxidase, and catalase) and glyoxalase enzymes (glyoxalase I and glyoxalase II) which led to the mitigation of oxidative damage indicated by reduced hydrogen peroxide, lipid peroxidation, and electrolyte leakage that ultimately improved growth, physiology, and yield of the bacterial inoculants rapeseed plants. When taken together, our results demonstrated the potential role of the plant probiotic bacteria, BRRh-4 and BTL-M2, in mitigating the Cd-induced damages in rapeseed plants.

## 1. Introduction

The rapid increase in metal pollution during the recent decades poses a severe threat to all living organisms. The primary reason behind this situation is the rapid growth of industries that indiscriminately excrete toxic metals/metalloids to nature and disrupt the equilibrium of the ecological components [1]. Among the metals/metalloids, cadmium (Cd) is the most toxic metal having a damaging effect on both plants and animals. The concentration of Cd beyond 5–10 mg g^−1^ dry weight of leaf creates phytotoxicity in the plants. Due to the hydrophilic nature of Cd, it could be rapidly accumulated through the roots and translocated easily to different tissues of plants, thus threatening the food chain of living organisms [2,3].

Cadmium toxicity is responsible for the alteration of the physiological processes of plants by creating obstacles in carbon fixation and photosynthetic pigment synthesis. However, Cd could not directly generate reactive oxygen species (ROS) through the Haber–Weiss reaction because of its redox-inactive nature [4]. Moreover, it interrupts the electron transport chain, antioxidant defense, and nutrient metabolism, which ultimately augment the generation of ROS, such as hydrogen peroxide (H_2_O_2),_ singlet oxygen (^1^O_2_), superoxide anion (O_2_^•−^), hydroxyl radical (^•^OH), etc. Overaccumulated ROS initiates the oxidation of the ultrastructure of biomolecules, e.g., carbohydrates, lipids, proteins, nucleic acids, etc., leading to the oxidative stress of the plants [5,6]. To combat the Cd-induced oxidative stress, plants evolved some avoidance techniques, viz., metal binding, metal chelation, vacuolar sequestration, compartmentalization, etc., to mitigate the pernicious effect of ROS [7,8].

Moreover, the extent of oxidative damage largely depends on the defensive mechanisms of plants under stress. The defensive mechanism of plants comprised of non-enzymatic (flavonoids, tocopherols, ascorbate, AsA; glutathione, GSH) and enzymatic (ascorbate peroxidase, APX; monodehydroascorbate reductase, MDHAR; dehydroascorbate reductase, DHAR; glutathione reductase, GR; glutathione peroxidase, GPX; catalase, CAT) antioxidants [5]. An efficient ROS scavenging through antioxidant defense maintains a proper redox balance and protects plants from oxidative stress. However, a steady increase of another cytotoxic compound, methylglyoxal (MG), under metal/metalloid toxicity is also liable for disrupting cellular organelles and producing plant mutations. However, the toxic effect of MG diminished with the activity of the glyoxalase system, which is composed of glyoxalase I (Gly I) and glyoxalase II (Gly II) enzymes that engaged in the detoxification of MG [9].

Removing heavy metal/metalloids from the contaminated sites through physicochemical remediation techniques is a high-priced and time-consuming process. In addition, these processes could lead to the accumulation of more complex subsidiary metal compounds in the soils [10]. Therefore, the auspicious role of metal-immobilizing plant growth-promoting rhizobacteria (PGPR) alleviates heavy metals pollution and stimulates growth and perseverance toward the toxic effects on plants [11]. The root zone of plants is rich in nutrients which accelerates microbial growth, and the root-associating rhizobacteria exude several metabolites that enhance the vicinity of stress tolerance of plants under harsh environmental conditions [12]. Recent advancements in the PGPR research have identified Cd-tolerant bacterial genera, such as *Bacillus* [13,14], and *Enterobacter* [15], *Serratia* [16], and *Pseudomonas* [17]. These microbes augment the production of phytohormones, antioxidant molecules, and some organic acids that enhance the resistance or tolerance capacity in plants. In addition to this, the PGPR regulates nitrogen fixation and phosphate solubilization, stabilizes pH, nutrient flow, and humidity under prolonged stress periods, and sustains the growth and development of plants [11,12]. Moreover, the use of PGPR as a bioremediation tool can be an effective way to decontaminate the Cd-contaminated agricultural fields.

Rapeseed (*Brassica campestris*) belongs to the Brassicaceae family, one of the widely cultivated oilseed crops in the subtropics and other regions of the world. All the parts of the plants are edible, containing a profound amount of nutrients and vitamins, and the oil contains a higher amount of healthy fatty acids that attract a lot for its cultivation. However, the plants of this Brassicaceae family are known as metal chelators due to their phytoextraction role [18]. Reports on *B.* *juncea* demonstrated that growth and oil content are adversely affected by metal toxicity, including Cd [19]. Moreover, beneficial growth-promoting bacteria are widely used in agriculture to enhance the growth and oil content of rapeseed sustainably [20,21].

Probiotic bacteria, *Paraburkholderia fungorum* and *Delftia* sp. establish plant-microbe association and produce phytohormones, antibiotics, and lytic enzymes, fix atmospheric nitrogen, solubilize soil minerals, induce systemic resistance to the host plants, and also have bioremediation potential [22,23,24,25]. The application of *P. fungorum* significantly increased the yield and fruit quality of strawberries in the field conditions [24]. Fruits from plants inoculated with the isolates *P. fungorum* BRRh-4 had significantly higher contents of phenolics, carotenoids, flavonoids, and anthocyanins than the non-treated control. Plants treated with this plant probiotic bacteria had no fungal disease infestation [24]. Biodegradation of fungicide and heavy metal removal by *Burkholderia fungorum* FM-2 and some species of *Paraburkholderia* spp. have been reported [25]. On the other hand, *Delftia* spp. are known to have heavy metal reducing and atmospheric nitrogen fixing abilities [26]. However, the roles of *P. fungorum* BRRh-4 and *Delftia* sp. BTL-M2 on plants’ tolerance to Cd-spiked soils are unknown. Therefore, we hypothesized that *P. fungorum* BRRh-4 and *Delftia* sp. BTL-M2 confer Cd tolerance in rapeseed. Thus, this study aimed to investigate the potentiality of *P. fungorum* and *Delftia* sp. in diminishing oxidative stress caused by Cd toxicity through upregulating defensive responses through coordinated actions of antioxidant defense and glyoxalase system in *B. campestris*.

## 2. Materials and Methods

### 2.1. Plant Materials, Experimental Conditions, and Treatments

Healthy and matured seeds of rapeseed (*B. campestris* cv. BARI Sarisha-14) were collected from Bangladesh Agriculture Research Institute (BARI), surface sterilized, and sown in well-prepared soil after applying the recommended dose of fertilizers [27] in the plastic pot (14 L). The plant growth-promoting probiotic bacteria, *P. fungorum* BRRh-4 and *Delftia* sp. BTL-M2 were isolated from the roots of rice and rice soils, respectively. The probiotic bacteria (BRRh-4 and BTL-M2) were cultivated separately in 500 mL nutrient broth (Merck, Germany) in conical flasks taking a single colony from actively growing bacterial culture plates that were maintained on a lab bench at ambient temperature (25 ± 2 °C) [24]. Then each flask was placed on a shaking incubator adjusted at 120 rpm and 25 °C for 72 h for bacterial growth in nutrient broth. The broth was centrifuged at 12,000× *g*, and the pellet was washed thrice with sterilized distilled water to remove nutrients. The bacterial pellet was suspended in water and diluted to a concentration of approx. 1 × 10^9^ CFU mL^−1^ and 5 × 10^8^ CFU mL^−1^ for *P. fungorum* BRRh-4 and *Delftia* sp. BTL-M2, respectively, according to the procedure of Rahman et al. [24]. Before sowing, seeds were treated with either *P. fungorum* BRRh-4 (ca. 1 × 10^9^ CFU mL^−1^) or *Delftia* sp. BTL-M2 (ca. 5 × 10^8^ CFU mL^−1^). After proper intercultural operations 14 days after sowing (DAS), sets of plants were subjected to two concentrations of Cd stress by adding 0.25 and 0.5 mM cadmium chloride (CdCl_2_). After growing for another 14 days in the Cd-supplemented conditions, growth, physiological, and biochemical parameters were recorded. The experiment was laid out in a completely randomized design (CRD) with three replications. One set of pots was used for estimating morphological, physiological, and biochemical parameters, and another group of plants was used to determine the yield and yield contributing parameters.

### 2.2. Measurement of Plant Height, SPAD Value, and Biomass Accumulation

Five plants were selected instinctively from each treatment and measured with a scale. The average value was expressed as centimeters (cm).

The reading of soil and plant analysis development (SPAD) was taken by using a SPAD meter (FT Green LLC, Wilmington, DE, USA) from five randomly selected fully expanded leaves to estimate the chlorophyll (Chl) content of the plants. Then the values were averaged to express the SPAD value.

At the junction of roots and shoots were excised and weighed in an electric balance for the estimation of fresh weight (FW). Hereafter, the roots and shoots were sun-dried, followed by oven dried at 80 °C for 72 h and weighed again for the dry weight (DW). Then, the mean value of the FW and DW of root and shoots were expressed as g plant^−1^.

### 2.3. Estimation of Relative Water Content

The relative water content (RWC) of the leaf was determined following the formula of Barrs and Weatherly [28]. The calculating formula is as follows:

$$\mathrm{Relative}\;\mathrm{water}\;\mathrm{content}\;\left(\%\right)=\frac{\mathrm{Fresh}\;\mathrm{weight}\;\left(\mathrm{FW}\right)-\mathrm{Dry}\;\mathrm{weight}\;\left(\mathrm{DW}\right)}{\mathrm{Turgid}\;\mathrm{weight}\;\left(\mathrm{TW}\right)-\mathrm{Dry}\;\mathrm{weight}\;\left(\mathrm{DW}\right)}\times 100$$

For estimating the FW, three leaf lamina were taken randomly from each treatment and weighed. Afterward, these leaves were placed in a Petri dish with adequate distilled water (dH_2_O) and covered with filter paper in a dark place for re-saturation. After 24 h of soaking in the dH_2_O, leaves were removed from the Petri dish, and excess water was wiped with tissue paper. Then turgid weight (TW) of the leaves was measured, followed by oven drying of those leaves at 80 °C for 72 h to determine the DW. After recording all the data, the RWC of the leaf was measured by using the above-mentioned formula.

### 2.4. Quantification of Proline Content

Proline (Pro) was determined according to the method of Bates et al. [29]. Fresh leaf of 0.5 g was taken and homogenized with 5 mL of 3% sulfosalicylic acid followed by centrifugation at 11,500× *g* for 15 min at 4 °C. After that, 1 mL of acid ninhydrin and 1 mL of glacial acetic acid were mixed with 1 mL of aliquot which was separated after centrifugation. This mixture was incubated in a water bath at 100 °C for 1 h. After cooling to room temperature, 4 mL of toluene was added to the mixture to separate the free Pro. The absorbance of the colored chromophore was measured spectrophotometrically at 520 nm using toluene as a blank. Finally, the Pro content was calculated with respect to a standard curve made with the known concentration of l-proline and expressed as µmol g^−1^ FW.

### 2.5. Estimation of Electrolyte Leakage

Electrolyte leakage (EL) of the leaf was estimated according to the method of Dionisio-Sese and Tobita [30] following the formula:

$$\mathrm{Electrolyte}\;\mathrm{leakage}\;\left(\%\right)=\frac{\mathrm{Initial}\;\mathrm{electrical}\;\mathrm{conductivity}\;\left({\mathrm{EC}}_{1}\right)\;}{\mathrm{Final}\;\mathrm{electrical}\;\mathrm{conductivity}\;\left({\mathrm{EC}}_{2}\right)}\times 100$$

Firstly, a harvested leaf of 0.5 g was taken into a Falcon tube and filled with 15 mL of dH_2_O. After that, each Falcon tube was incubated in the water bath at 40 °C for 1 h. The initial electrical conductivity (EC_1_) was measured with an electrical conductivity (EC) meter (HI-993310, Hanna, RI, USA) after cooling at room temperature. These Falcon tubes were incubated in an autoclave at 121 °C for 20 min, then cooled in an ice bath to room temperature. Final conductivity (EC_2_) was taken again with the EC meter, and the calculation of EL of the leaf was done following the above-mentioned formula.

### 2.6. Determination of Stress Indicators

For estimating malondialdehyde (MDA) content, 0.5 g of the fresh leaf was mashed with 3 mL of 5% (*w*/*v*) trichloroacetic acid (TCA) using chilled mortar and pestle and centrifuged for 15 min at 11,500× *g*. Then the 1 mL of the obtained aliquot after centrifugation was consolidated with 4 mL of thiobarbituric acid (TBA) reagent that contained 0.5% of TBA dissolved in the 20% TCA according to the procedure of Heath and Packer [31]. After being heated at 95 °C for 30 min in a water bath, the reaction mixture was promptly chilled in an ice bath. The absorbance of the colored chromophore was observed at 532 nm and adjusted for non-specific absorbance at 600 nm. After using the extinction coefficient of 155 mM^−1^ cm^−1^, the amount of MDA content was estimated and expressed as nmol g^−1^ FW.

The hydrogen peroxide (H_2_O_2_) content was measured using the technique by Yu et al. [32]. At 4 °C, 0.5 g of leaf tissue was macerated with mortar and pestle in 5% TCA and centrifuged for 15 min at 11,500× *g* to obtain a clear aliquot. Afterward, 1 mL of the obtained aliquot was fused with 1 mL of potassium-phosphate (K-P) buffer pH 7 (10 mM) and 1 mL of potassium iodide (1 mM), then the mixture was kept in the dark for 1 h. In order to quantify the H_2_O_2_, the optical density of the mixture was taken at 390 nm by spectrophotometer, and the calculated value of H_2_O_2_ content was expressed as nmol g^−1^ FW.

### 2.7. Extraction and Quantification of Protein Content and Enzymes Activity Assay

Fresh leaf (0.5 g) was macerated in a pre-cooled mortar pestle using 1 mL of extraction buffer, which contained 50 mM K-P buffer (pH 7.0), 100 mM potassium chloride (KCl), 1 mM L-ascorbic acid (Asc), 5 mM β-mercaptoethanol, and 10% (*w*/*v*) glycerol, and subjected for centrifugation at 4 °C (11,500× *g*, 10 min). Then, the obtained aliquot was used to determine protein concentration and enzyme activity [33].

The protein content was determined according to the method of Bradford [34] using bovine serum albumin (BSA) as a protein standard. At first, the Bradford reagent was prepared with the Coomassie brilliant blue (G-250), ethanol (100%), phosphoric acid (85%), and dH_2_O. Then, 5 µL of the aliquot and 5 mL of Bradford reagent were incorporated, and the absorbance of the mixture was observed at 595 nm by spectrophotometer. Afterward, the absorbance of the unknown sample was plotted against a standard curve prepared from the known concentration of BSA, and the protein concentration was determined.

The APX (EC: 1.11.1.11) activity was measured using Nakano and Asada’s [35] technique. In a final volume of 0.7 mL, the reaction buffer solution contained K-P buffer (50 mM, pH 7.0), Asc (0.5 mM), H_2_O_2_ (0.1 mM), ethylenediaminetetraacetic acid (EDTA; 0.1 mM), and enzyme extract. The H_2_O_2_ was used to initiate the reaction, and an extinction coefficient of 2.8 mM^−1^ cm^−1^ was used to assess activity by measuring the decline in absorbance for 1 min at 290 nm spectrophotometrically.

The MDHAR (EC: 1.6.5.4) activity was determined by the method of Hossain et al. [36]. The reaction mixture contained Tris–HCl buffer (50 mM, pH 7.5), nicotinamide adenine dinucleotide phosphate (NADPH; 0.2 mM), Asc (2.5 mM), ascorbate oxidase (AO; 0.5 U), and the total volume of the enzyme mixture was 0.7 mL. After the addition of AO to the mixture, the reaction was initiated. The ascorbate’s changing activity was determined at 340 nm by a spectrophotometer for 1 min using an extinction coefficient of 6.2 mM^−1^ cm^−1^.

The DHAR (EC: 1.8.5.1) activity was assessed using the Nakano and Asada method [35]. The K-P buffer (50 mM, pH 7.0), reduced glutathione (GSH; 2.5 mM), and dehydroascorbate (DHA; 0.1 mM) were present in the reaction buffer. The enzyme solution was added to the reaction buffer to initiate the reaction. In using an extinction value of 14 mM^−1^ cm^−1^, the activity was determined from the change in absorbance for 1 min at 265 nm spectrophotometrically.

The GR (EC: 1.6.4.2) activity was assessed using the Hasanuzzaman et al. [33] technique. In a final volume of 1 mL, the reaction mixture contained K-P buffer (0.1 M, pH 7.8), EDTA (1 mM), oxidized glutathione (GSSG; 1 mM), NADPH (0.2 mM), and enzyme solution. The GSSG was used to start the reaction, and for 1 min, the NADPH oxidation-related drop in absorbance at 340 nm was observed in a spectrophotometer. In utilizing an extinction value of 6.2 mM^−1^ cm^−1^, the activity was computed.

Utilizing H_2_O_2_ as a substrate, the GPX (EC: 1.11.1.9) activity was assessed as per Elia et al. [37] instruction. The reaction mixture included sodium-phosphate (Na-P) buffer (100 mM, pH 7.5), EDTA (1 mM), sodium azide (NaN_3_; 1 mM), NADPH (0.12 mM), GSH (2 mM), GR (1 U), and H_2_O_2_ (0.6 mM). Here, H_2_O_2_ was added to start the reaction. In using the extinction coefficient of 6.62 mM^−1^ cm^−1^, the activity of NADPH oxidation was measured at 340 nm for 1 min spectrophotometrically.

The CAT (EC: 1.11.1.6) activity was assessed by observing the drop in absorbance at 240 nm for 1 min brought by the breakdown of H_2_O_2_. In a final volume of 0.7 mL, the reaction mixture contained K-P buffer (50 mM, pH 7.0), H_2_O_2_ (15 mM), and enzyme solution. After adding H_2_O_2_ to the reaction mixture, the enzyme started the reaction and computed the activity by using 39.4 M^−1^ cm^−1^ as an extinction coefficient [33].

The Gly I (EC: 4.4.1.5) enzyme assay mixture, which had a final volume of 0.7 mL, was composed of K-P buffer (100 mM, pH 7.0), magnesium sulfate (MgSO_4_; 15 mM), GSH (1.7 mM), and MG (3.5 mM). The addition of MG triggered the process, and the rise in absorbance was observed at 240 nm for 1 min spectrophotometrically, and utilizing an extinction value of 3.37 mM^−1^ cm^−1^, the activity was computed [33].

The production of GSH at 412 nm for 1 min in a spectrophotometer was used to measure the activity of Gly II (EC: 3.1.2.6) following the procedure of Principato et al. [38]. In a final volume of 1 mL, the reaction mixture contained Tris-HCl buffer (100 mM, pH 7.2), 5,5-dithio-bis(2-nitrobenzoic acid) (DTNB; 0.2 mM), and *S*-_D_-lactoylglutathione (SLG; 1 mM). The SLG was used to initiate the reaction, and the activity was determined using the 13.6 mM^−1^ cm^−1^ extinction coefficient.

### 2.8. Statistical Analysis

The parameters were studied from three replications, and the results were presented as mean values with standard deviations (±SD). The significant differences among the treatments were statistically analyzed by using the CoStat v.6.400 computer-based software (Co-Hort Software, Monterey, CA, USA) after applying Tukey’s HSD test at *p* ≤ 0.05 level of significance [39]. Correlation analysis was done using Origin Pro 2022 (OriginLab, Northampton, MA, USA).

## 3. Results

### 3.1. Growth Parameters and Photosynthetic Activity

**Figure 1:**
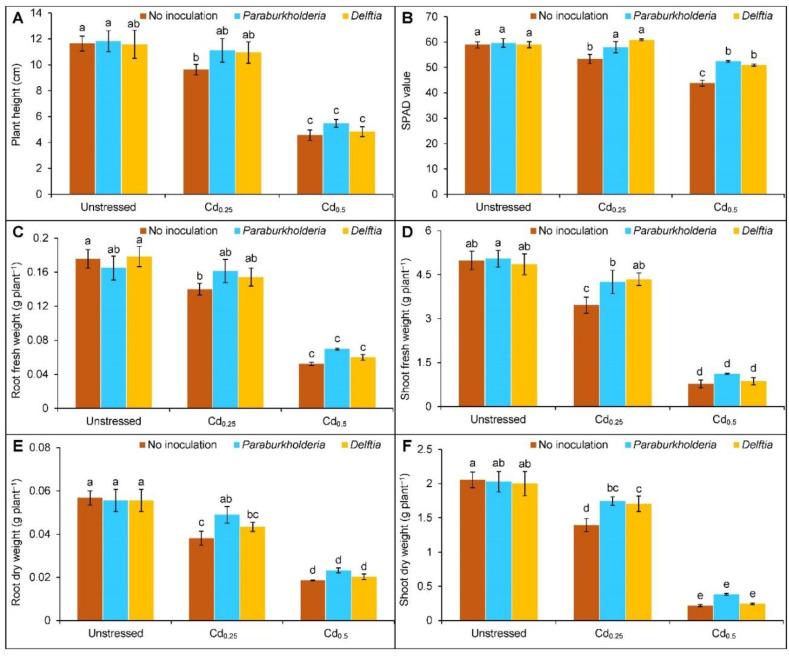
Effect of *Paraburkholderia fungorum* and *Delftia* sp. on the (**A**) plant height, (**B**) SPAD value, (**C**) root fresh weight, (**D**) shoot fresh weight, (**E**) root dry weight, and (**F**) shoot dry weight of rapeseed under different levels of CdCl_2_. Here, Cd_0.25_ and Cd_0.5_ indicated 0.25 and 0.5 mM CdCl_2_, respectively. Different letters on the bars indicate statistical significance at 5% levels of probability following Tukey’s HSD test and the mean (±SD) values obtained from three replications.

Plants treated with Cd_0.25_ and Cd_0.5_ reduced the plant height by 17 and 61%, respectively, compared to the controls (Figure 1A). However, *P. fungorum* and *Delftia* sp. inoculation did not enhance the height at Cd_0.25_- and Cd_0.5_-treated plants.

Compared to controls, the SPAD value was reduced by 9% in Cd_0.25_- and 26% in Cd_0.5_-stressed plants (Figure 1B). The supplementation of *P. fungorum* enhanced the value by 9 and 20%, respectively. In contrast, the value increased by 14 and 16% with the *Delftia* sp., respectively, in the Cd_0.25_- and Cd_0.5_-stressed plants compared to the corresponding Cd treated plants.

Under Cd toxicity, biomass accumulation was also suppressed. Under Cd_0.25_, the root FW, shoot FW, root DW, and shoot DW were decreased by 20, 31, 33, and 32%, respectively. On the contrary, the reduction was 70, 85, 67, and 89%, respectively, when treated with Cd_0.5_ (Figure 1C–F). In the *P. fungorum* inoculated Cd_0.25_-stressed plants, the shoot FW (23%), root DW (28%), and shoot DW (25%) were increased compared to the uninoculated Cd-treated plants. Similarly, the *Delftia* sp. inoculation only enhanced the shoot FW (26%) and DW (22%) in the Cd_0.25_-treated plants, respectively, compared to the uninoculated Cd-treated plants. At the Cd_0.5_-treated plants, the inoculation of either *P. fungorum* or *Delftia* sp. did not significantly increase the FW and DW of roots and shoots.

### 3.2. Relative Water Content and Proline Accumulation

**Figure 2:**
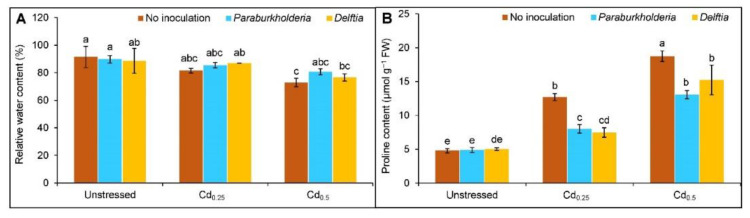
Effect of *Paraburkholderia fungorum* and *Delftia* sp. on the (**A**) relative water content (RWC) and (**B**) proline (Pro) content of rapeseed under different levels of CdCl_2_. Here, Cd_0.25_ and Cd_0.5_ indicated 0.25 and 0.5 mM CdCl_2_, respectively. Different letters on the bars indicate statistical significance at 5% levels of probability following Tukey’s HSD test and the mean (±SD) values obtained from three replications.

Upon exposure to Cd stress, the RWC of leaf reduced by 19% in Cd_0.5_-stressed plants in comparison to controls, whereas no change was observed under Cd_0.25_ (Figure 2A). However, the leaf RWC did not enhance significantly with the inoculation of *P. fungorum* and *Delftia* sp. in the Cd_0.25_- and Cd_0.5_-stressed plants.

A steady increase of Pro content by 165 and 292% at Cd_0.25_ and Cd_0.5_, respectively, compared to controls (Figure 2B). The *P. fungorum* decreased the Pro content by 37 and 41%, while *Delftia* sp. decreased it by 30 and 19%, respectively, in plants treated with Cd_0.25_ and Cd_0.5_ compared to the Cd alone.

### 3.3. Oxidative Stress Indicators

**Figure 3:**
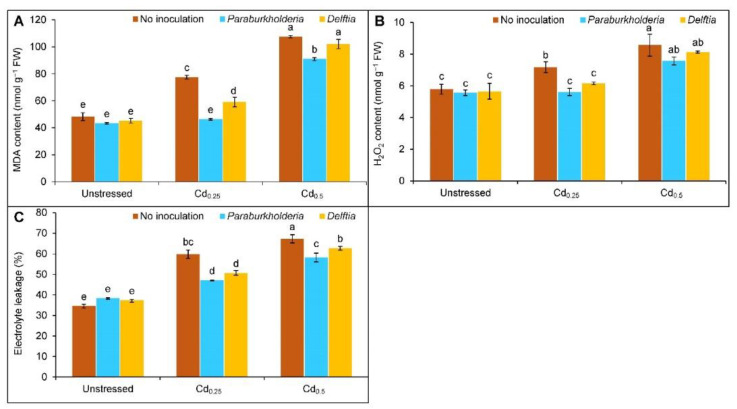
Effect of *Paraburkholderia fungorum* and *Delftia* sp. on the content of (**A**) malondialdehyde (MDA), (**B**) hydrogen peroxide (H_2_O_2_), and (**C**) electrolyte leakage (EL) of rapeseed under different levels of CdCl_2_. Here, Cd_0.25_ and Cd_0.5_ indicated 0.25 and 0.5 mM CdCl_2_, respectively. Different letters on the bars indicate statistical significance at 5% levels of probability following Tukey’s HSD test and the mean (±SD) values obtained from three replications.

Oxidative stress indicators, the MDA content increased by 60 and 122% at Cd_0.25_ and Cd_0.5_, while the increment of H_2_O_2_ content was found by 24 and 48%, respectively, compared to control plants. Similarly, the EL was also upgraded by 25 and 33% at Cd_0.25_ and Cd_0.5_ stress, respectively. (Figure 3A–C). However, the *P. fungorum* reduced the MDA content by 40% in Cd_0.25_- and 15% in Cd_0.5_-stressed plants, respectively, compared to the corresponding Cd treated alone. In contrast, the *Delftia* sp. only reversed the MDA content by 24% in Cd_0.25_-stressed plants but showed no improvement at Cd_0.5_ (Figure 3A). The H_2_O_2_ content decreased by 22 and 14%, respectively, with the *P. fungorum* and *Delftia* sp. inoculation at Cd_0.25_ compared to Cd alone (Figure 3B). Conversely, the bacterial inoculation did not decrease the H_2_O_2_ content significantly at Cd_0.5_ stress. A similar reduction of the EL was also observed with the *P. fungorum* (13 and 9%) and *Delftia* sp. (9 and 5%) in plants with Cd_0.25_ and Cd_0.5_ stress (Figure 3C).

### 3.4. Activities of Antioxidant Enzymes

**Figure 4:**
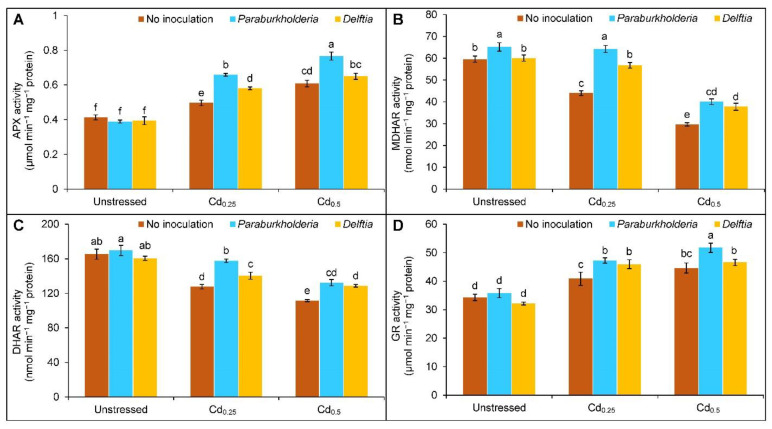
Effect of *Paraburkholderia fungorum* and *Delftia* sp. on the activities of (**A**) ascorbate peroxidase (APX), (**B**) monodehydroascorbate reductase (MDHAR), (**C**) dehydroascorbate reductase (DHAR), and (**D**) glutathione reductase (GR) of rapeseed under different levels of CdCl_2_. Here, Cd_0.25_ and Cd_0.5_ indicated 0.25 and 0.5 mM CdCl_2_, respectively. Different letters on the bars indicate statistical significance at 5% levels of probability following Tukey’s HSD test and the mean (±SD) values obtained from three replications.

Cadmium stress augmented the activity of APX (20 and 47%) and GR (19 and 30%), whereas it suppressed the MDHAR (26 and 50%) and DHAR (23 and 33%) activities at Cd_0.25_ and Cd_0.5_, respectively, in comparison to controls (Figure 4A–D). The APX activity was further enhanced by 33 and 26% with the *P. fungorum* in the plants treated with Cd_0.25_ and Cd_0.5_ stress, respectively. *Delftia* sp., on the other hand, only improved the activity of APX by 17% with Cd_0.25_-treated plants compared to Cd only (Figure 4A). Compared to the uninoculated Cd stressed plants, the *P. fungorum* and *Delftia* sp. increased the MDHAR activity by 46 and 29%, and by 35 and 27%, respectively, in the plants at Cd_0.25_ and Cd_0.5_ stress (Figure 4B). Likewise, the *P. fungorum* upgraded the activity of DHAR by 24 and 10% and GR by 16 and 13%, respectively, in the Cd_0.25_ and Cd_0.5_ stress in comparison to Cd treated alone. However, the *Delftia* sp. also augmented the DHAR activity by 10% in Cd_0.25_ and 15% in Cd_0.5_ and uplifted the GR activity only at Cd_0.25_ stress by 13% compared to corresponding Cd stressed only (Figure 4C,D).

**Figure 5:**
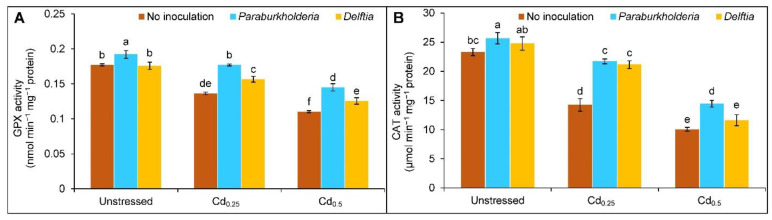
Effect of *Paraburkholderia fungorum* and *Delftia* sp. on the activity of (**A**) glutathione peroxidase (GPX) and (**B**) catalase (CAT) of rapeseed under different levels of CdCl_2_. Here, Cd_0.25_ and Cd_0.5_ indicated 0.25 and 0.5 mM CdCl_2_, respectively. Different letters on the bars indicate statistical significance at 5% levels of probability following Tukey’s HSD test and the mean (±SD) values obtained from three replications.

The activity of GPX was declined by 23 and 38% in the plant treated with Cd_0.25_ and Cd_0.5_, respectively, compared to controls. However, the GPX activity was reversed with *P. fungorum* (30 and 32%) and *Delftia* sp. (15 and 14%) at Cd_0.25_ and Cd_0.5_ compared to Cd only (Figure 5A). A similar trend was observed in the case of CAT activity under Cd stress. However, the activity was enhanced with *P. fungorum* and *Delftia* sp. by 52 and 48% at Cd_0.25_, whereas at Cd_0.5_, it was increased by 44% only with the *P. fungorum*, compared to Cd treated alone (Figure 5B).

### 3.5. Activities of Glyoxalase Enzymes

**Figure 6:**
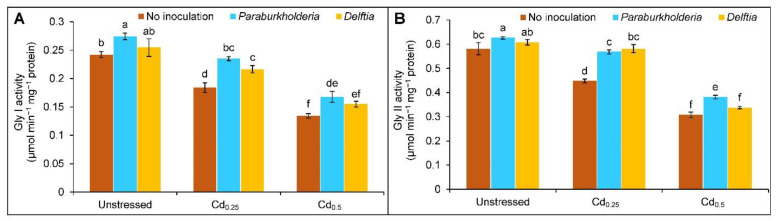
Effect of *Paraburkholderia fungorum* and *Delftia* sp. on the activities of (**A**) glyoxalase I (Gly I) and (**B**) glyoxalase II (Gly II) of rapeseed under different levels of CdCl_2_. Here, Cd_0.25_ and Cd_0.5_ indicated 0.25 and 0.5 mM CdCl_2_, respectively. Different letters on the bars indicate statistical significance at 5% levels of probability following Tukey’s HSD test and the mean (±SD) values obtained from three replications.

Both Gly I and Gly II activities were decreased with the gradual increase of Cd doses. The activity of Gly I and Gly II declined by 24 and 44% and by 23 and 47% in the plants treated with the Cd_0.25_ and Cd_0.5_ stress, respectively, compared to the controls. However, the augmentation of the Gly I activity by 28 and 25% and Gly II activity by 27 and 24% in the *P. fungorum* inoculated plants treated with the Cd_0.25_ and Cd_0.5_ stress, respectively, in comparison to the corresponding Cd alone. In contrast, the *Delftia* sp. inoculation only enhanced the Gly I (18%) and Gly II (30%) activities at Cd_0.25_, respectively, compared to the Cd alone, but no changes were observed at Cd_0.5_-stressed plants (Figure 6A,B).

### 3.6. Yield Parameters

**Figure 7:**
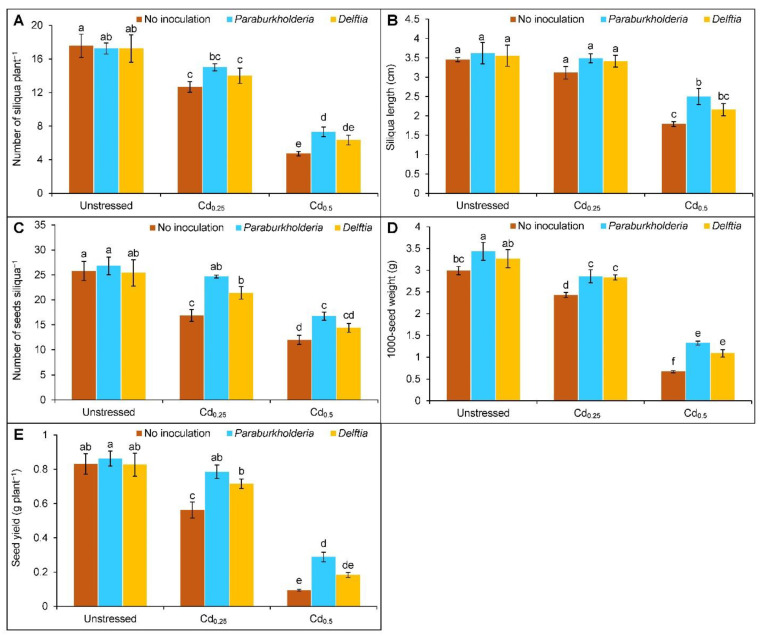
Effect of *Paraburkholderia fungorum* and *Delftia* sp. on the (**A**) number of siliqua plant^−1^, (**B**) siliqua length, (**C**) number of seeds siliqua^−1^, (**D**) 1000-seed weight, and (**E**) seed yield plant^−1^ of rapeseed under different levels of CdCl_2_. Here, Cd_0.25_ and Cd_0.5_ indicated 0.25 and 0.5 mM CdCl_2_, respectively. Different letters on the bars indicate statistical significance at 5% levels of probability following Tukey’s HSD test and the mean (±SD) values obtained from three replications.

The toxicity of Cd considerably declined the yield parameters of the rapeseed plants. The number of siliqua plant^−1^ was decreased by 28 and 73% at Cd_0.25_ and Cd_0.5_, respectively, compared to the controls. However, the *P. fungorum* only increased the number of siliqua plant^−1^ in the Cd_0.5_ treated plants by 55% compared to the corresponding uninoculated Cd stressed only. The siliqua length declined at Cd_0.5_ by 48% compared to the controls. However, the *P. fungorum* increased the siliqua length by 40% at Cd_0.5_ stress compared to the respective Cd treated alone. Similarly, the number of seeds siliqua^−1^ (35 and 53%), 1000-seed weight (19 and 77%), and seed yield plant^−1^ (32 and 89%) were declined when treated with Cd_0.25_ and Cd_0.5_ stress, respectively, in comparison to the control plants. However, the *P. fungorum* improved the number of seeds siliqua^−1^ by 46 and 39% at Cd_0.25_ and Cd_0.5_, respectively. In contrast, the *Delftia* sp. only increased it at Cd_0.25_ by 27% compared to the Cd alone. Likewise, the *P. fungorum* enhanced 1000-seed weight by 18 and 97%, and *Delftia* sp. by 17 and 62%, respectively, in plants treated with the Cd_0.25_ and Cd_0.5_ stress compared to the respective Cd treated alone. Furthermore, the seed yield plant^−1^ also enhanced with the *P. fungorum* by 40 and 204%, respectively, at Cd_0.25_ and Cd_0.5_, whereas the *Delftia* sp. only increased the seed yield plant^−1^ at Cd_0.25_ treated plants by 27% in comparison to the corresponding uninoculated Cd alone (Figure 7A–E).

### 3.7. Correlation Analysis among the Parameters

**Figure 8:**
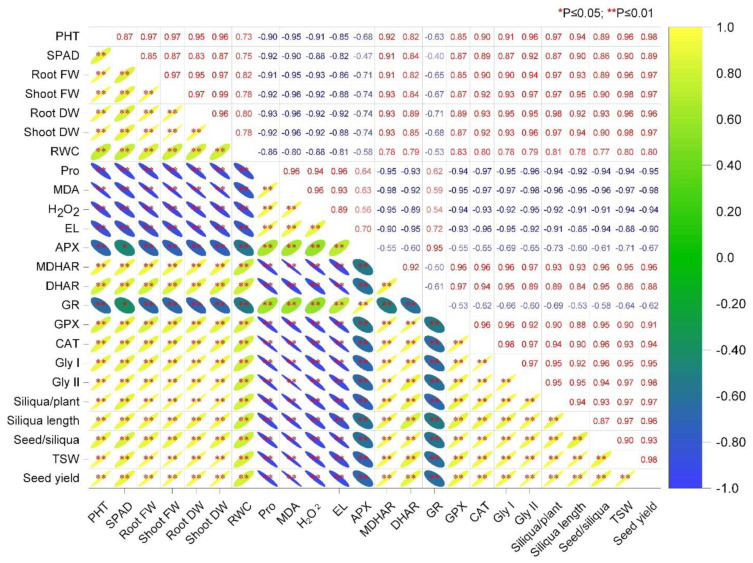
Correlation matrix among the different parameters of rapeseed as affected cadmium stress. Here, PHT—plant height; SPAD—soil and plant analysis development value; FW—fresh weight; DW—dry weight; RWC—relative water content; Pro—proline; MDA—malondialdehyde; H_2_O_2_—hydrogen peroxide; EL—electrolyte leakage; APX—ascorbate peroxidase; MDHAR—monodehydroascorbate reductase; DHAR—dehydroascorbate reductase; GR—glutathione reductase; GPX—glutathione peroxidase; CAT—catalase; Gly I—glyoxalase I; Gly II—glyoxalase II; TSW—thousand seed weight.

The correlation analysis among the different parameters is depicted in Figure 8. From the matrix, it can be stated that the parameters of oxidative stress indicators, such as MDA, H_2_O_2_, and EL, are negatively correlated with the MDHAR, DHAR, GPX, CAT, Gly I, and Gly II activities. In contrast, the APX and GR activities are positively interlinked. Thus, the plant growth, biomass production, and yield components are negatively affected by the Cd-induced oxidative stress indicators (Figure 8).

## 4. Discussion

Cadmium ubiquitously presents in nature which is a highly phytotoxic compound that may cause significant yield reductions and is also carcinogenic for animals and humans [3]. The toxic effect of Cd leads to growth inhibition and alters different biochemical processes, viz., photosynthetic activities, stomatal movements, carbon dioxide fixation, etc. [8]. Under Cd stress, the accumulation of iron [Fe(III)] becomes limited, thus inhibiting the activity of Fe(III) reductase, which abruptly affects photosynthesis and distorts the growth and biomass accumulation of the plants [8]. The retardation of growth attributes and leaf chlorosis due to impaired photosynthesis were also reported in the Cd-stressed *Brassica napus* [15], *Zea mays* [16], and *Solanum nigrum* [14] seedlings. In the current study, we demonstrated that two rice-associated growth-promoting bacteria, *P. fungorum* BRRh-4 and *Delftia* sp. BTL-M2 confer cadmium tolerance in rapeseed (*B. campestris*) plants, likely through modulating the antioxidant defense and glyoxalase systems in the plant cells. Although growth and yield-promoting effects of *P. fungorum* and *Delftia* sp. have been reported [23,24,26], this study, for the first time, demonstrated that these rice probiotic bacteria enhance plant tolerance to a heavy metal via strengthening the antioxidant defense system.

Amelioration of Cd toxicity by metal-resistant endophytic bacteria by immobilizing or modifying the availability of metals and enhancing the regulation of the essential nutrient uptake to the plants by synthesizing exopolysaccharides (EPS), siderophores production, acidification, or solubilizing phosphates have previously been reported [40]. Notably, plant growth (root length, shoot length, FW, and DW) and SPAD value were retrieved with supplementation of *Enterobacter* sp. in *B. napus* under Cd stress, as reported by Saeed et al. [15]. Under Cd stress, the *Enterobacter*-inoculated plants showed improved photosynthetic rate (P*_n_*), transpiration rate (T*_r_*), and stomatal conductance (g*_s_*), thus enhancing the growth and biomass production of *B. napus* [15]. Likewise, Tanwir et al. [16] demonstrated inoculation of *Serratia* sp. incremented plant height, Chl content, and biomass production of the Cd-affected maize plants compared to the uninoculated plants. Moreover, Gupta et al. [41] confirmed that such growth promotion by inoculating *Bacillus* sp. bacteria was attenuated by increased accumulation of indole acetic acid (IAA), phosphate solubilization, and siderophore production under salt stress. Furthermore, Chi et al. [14] evaluated the gene expression of *IRT1*, *HMA*, and *PDR2* that regulate Cd uptake, transport, and detoxification. They reported that in the Cd-stressed *S. nigrum*, the expression of *IRT1* increased and suppressed the *HMA,* which led to a limit of the Cd deposition in the roots rather than shoots [14]. In addition to these, the microbe triggers phytohormone production and facilitates in the Cd detoxification system [42,43]. Chi et al. [14] also reported similar stimulation of the plant growth and root development by stimulating the IAA accumulation in Cd-spiked *S. nigrum* plants with the inoculation of *B. megaterium.* These results confirmed the PGPR-induced resistance toward the abrupt environmental conditions and improved growth and biomass production supported by the present study with the rhizobacteria, *P. fungorum*, and *Delftia* sp. inoculated rapeseed plants under Cd stress.

Osmotic balance is disrupted under metal/metalloid toxicity due to secondary effects. Therefore, plants accumulate secondary metabolites such as Pro to cope with the changing water balance, which is an effective osmoprotectant and aids in chelating metal ions [44]. In the current study, under Cd stress, the RWC of rapeseed leaves is reduced in a dose-dependent manner which is in congruence with the previous findings of Cd-stressed *B. napus* [15]. However, in the present study, cellular dehydration has been ameliorated with the addition of *P. fungorum* and *Delftia* sp. in the Cd-stressed rapeseed plants. Likewise, bacterial inoculation of *Enterobacter* sp. enhanced the water content of *B. napus,* as reported by Saeed et al. [15] under Cd toxicity. Reports on the endophytic bacteria *Burkholderia phytofirmans* showed that water stress was alleviated in the salt-stressed *Chenopodium quinoa* plants [45]. Thus, to adjust the water balance, biosynthesis of osmolytes such as Pro is a key strategy to negate the osmotic shock under Cd stress [46]. The higher accumulation of Pro in response to Cd in the current investigation is supported by the previous findings [47,48]. However, in response to Cd stress, the *P. fungorum* and *Delftia* sp. inoculated rapeseed plants and enhanced water content mitigated Pro accumulation in the Cd stressed plants. Moreover, Lastochkina et al. [49] reported that *B. subtilis* inoculation in response to salinity stress and Pro accumulation was reduced in *Phaseolus vulgaris* under salinity. Similar retardation of Pro content was also found with the endophytic inoculation of *B. subtilis* in the salt-affected *Triticum aestivum* plants [50]. Moreover, microbes accelerated the production of EPS which enhanced adherence of soil particles and also aided in promoting macropore production to improve the soil porosity and aeration under stressful environments, thus maintaining osmotic adjustment [51].

Upon exposure to Cd stress, excessive generation of ROS such as H_2_O_2_, ^1^O_2_, and O_2_^•−^, etc., leads to oxidative stress that distorts proteins, lipids, amino acids, and nucleic acids. The MDA content is a prime indicator of oxidative stress produced due to membrane damage resulting from lipid peroxidation [5]. Marked increment of H_2_O_2_ and MDA in a dose-dependent manner, as well as EL, observed by Cd stress in the current investigation. ROS degrades the activity of the enzyme that stabilizes lipids and regulates membrane damage. However, the PGRR boosted plants’ stress tolerance by retaining water status, uptaking essential nutrients, accumulating metabolites, and augmenting the defensive responses [52]. In a previous report on Cd-spiked *B. juncea* plants, the bacterial inoculants *Serratia* sp. decremented the MDA and EL levels by triggering the internal immune system [53]. Moreover, the antioxidant enzyme activities (SOD, CAT, and POD) were uplifted in the *G. max* with *B. cereus* inoculation that detoxifies ROS [13]. Similarly, inoculation of *Sphingobium yanoikuyae* diminished H_2_O_2_ and MDA accumulation in the Cd-treated *Salix matsudana* plants and sustained the membrane structure [54]. Likewise, Chi et al. [14] reported *Bacillus* sp. inoculation combat the ROS damage and degree of lipid peroxidation by augmenting the antioxidant enzyme activities. However, the *P. fungorum* and *Delftia* sp. inoculated rapeseed plants showed reduced Cd-induced oxidative stress indicators (MDA, H_2_O_2_, and EL) that displayed the potential role of endophytes in alleviating oxidative stress in plants.

To dissipate the excess generation of ROS and its damaging effect on the cellular organelles, plants activate their defensive system consisting of several antioxidant enzymes, such as APX, MDHAR, DHAR, GR, GPX, and CAT, thus helping to sustain the stressed period. Moreover, four vital antioxidant enzymes are directly involved in the detoxification of ROS in the AsA-GSH cycle. In the absence of CAT in the chloroplast, APX scavenges ROS along with the GR and maintains redox homeostasis [5]. In the Cd-stressed plants, the APX activity was enhanced, indicating H_2_O_2_ scavenging through the production of H_2_O, further accelerating the activity of APX was observed in the bacteria-inoculated plants. The APX-regulated redox homeostasis is initiated with the catalytic reaction in the AsA-GSH cycle, where MDHA is produced and yielded into DHA [55]. Moreover, the APX activity is augmented in the Cd-spiked *Medicago sativa* with the *Rhizobium* sp. and *Pseudomonas* sp. inoculation [17]. Chi et al. [14] reported the enhancement of APX activity with the endophytic inoculation of *Bacillus* strains in the Cd-stressed *S. nigrum* plants. Likewise, augmentation of the APX activity was also reported with the *Streptomyces* sp. and *Nocardiopsis* sp. inoculated *Sorghum bicolor* plants under Cd stress [3]. The MDHAR revived the AsA regeneration from MDHA by using NADPH and lowered the ROS-induced cellular damages [9]. In the current study, the endophyte inoculation improved MDHAR activity, increasing the AsA production and thus enhancing stress tolerance. Similarly, the *Bacillus* inoculants *Abelmoschus esculentus* plants showed elevated MDHAR activity under drought stress [56]. The DHAR is a GSH-based monomeric enzyme engaged in GSH-dependent DHA recycling [57]. Further regeneration of the GSH from GSSG is mediated by the activity of GR. Higher amounts of GSH help the metal chelation and engage in the MG detoxification system. Under Cd stress, the activities of DHAR and GR were increased, and further acceleration was observed in the bacteria-inoculated plants in the present investigation. Thus, the enhanced activity of antioxidant enzymes in the AsA-GSH cycle enhances ROS scavenging and aids in redox maintenance in the plants [9]. The increased activity of DHAR was reported by Puthiyottil and Akkara [56] in the drought-affected *A. esculentus*. Moreover, the GR activity was also enhanced in the *Cicer arietinum* plants upon inoculation with the endophytic *B. subtilis* under salinity [58]. Other antioxidant enzymes (GPX and CAT) are also engaged in the ROS scavenging system in the plants. The GPX utilized GSH to detoxify H_2_O_2_ and convert it to H_2_O. Likewise, CAT detoxifies H_2_O_2_ through the production of H_2_O and O_2_. Thus, GPX and CAT play a crucial role in the ROS detoxification system [5]. The activity of GPX was enhanced in the *Medicago sativa* with the *Rhizobium* sp. and *Pseudomonas* sp. inoculation under Cd toxicity [17]. Previously, in the copper-stressed *S. lycopersicum*, the activity of GPX was also augmented with the *B. amyloliquefaciens* [59]. Moreover, increased activity of CAT in the *S. matsudana* with the endophytic *S. yanoikuyae* is also found under Cd-stressed plants [54]. The activity of GPX and CAT was also augmented in the current study with probiotic bacteria inoculation. Thus, it can be stated that PGPB enhanced the defense responses of plants by participating in the ROS scavenging system and inducing plant tolerance.

Methylglyoxal is a cytotoxic compound produced in plants as a derivative of biochemical pathways under stress. In order to protect the cellular organelles from this glycolytic effect of MG, plants are equipped with an MG detoxification system which consists of Gly I and Gly II enzymes [9]. These enzymes degrade MG and convert it to a non-toxic form in two ways. First, the utilization of GSH by Gly I for the conversion of MG to SLG, and finally, SLG is transformed into D-lactate by the action of Gly II [9]. Under Cd stress, the activities of Gly I and Gly II were reduced remarkably in the current investigation, signifying lesser detoxification of MG-induced oxidative stress in the rapeseed plants. Such declination of glyoxalase enzyme activity was reported in different plants [33,60]. The overproduced MG oxidizes GSH to GSSG and disturbs various physiological pathways [61]. However, the *P. fungorum* and *Delftia* sp. showed enhanced activities of Gly I and Gly II in rapeseed plants, which might have upregulated the MG detoxification and improved GSH levels under Cd stress. Moreover, GSH also acts as a metal chelator by producing derivatives of metal complexes and storing them in the vacuole as inert matter [62].

Upon Cd stress, plant growth and reproductive development are significantly affected due to the damaging effects of oxidative stress and nutrient deficiencies, thus causing subsequent yield retardation [63,64]. In this investigation, yield contributing parameters were abruptly affected in the Cd-stressed rapeseed plants, which were correlated with the seed yield plant^−1^. The application of endophytic bacteria restored plant physiological attributes, such as P*_n_*, T*_r_*, and internal carbon dioxide concentration (C*_i_*), which enhanced the plant growth under an abrupt environment and ultimately improved yield parameters [65]. Supplementation of *Enterobacter* sp. in the *Pisum sativum* improved 100-seed weight under Cd stress reported by Naveed et al. [63]. In the Cd-stressed *P. sativum* plants, the bacteria enhanced the P*_n_*, T*_r_*, and photosynthesis which improved the growth and yield of the plants [63]. Similarly, under Cd stress, the panicle numbers of rice have been increased with the Cd-resistant bacteria *B. koreensis*. In contrast, the 1000-grain weight was not improved in the bacterial-inoculated plants but reduced the Cd deposition in rice grains [66]. Moreover, Kumar et al. [64] also reported that *B. pumilus* improved grain weight in salt-stressed rice. Such improvement was attributed to the increment of photosynthetic activities that enhanced carbohydrate translocation and photoassimilate production in the Cd-affected plants with bacterial inoculation. Furthermore, the quality parameters, such as the contents of protein, fat, ash, and fiber of *P. sativum,* were augmented with the inoculation of *Enterobacter* sp. under Cd toxicity [63]. Overall, the PGPB inoculation remediates Cd toxicity by improving biochemical and physiological attributes to improve the plant’s growth and development, which finally recovered the yield contributing components as well as the yield of rapeseed plants.

In summary, the application of *P. fungorum* BRRh-4 and *Delftia* sp. BTL-M2 conferred cadmium tolerance in rapeseed. The Cd-treated plants showed reduced plant growth, biomass accumulation, impaired photosynthetic activity along with reduced RWC. In contrast, increased Pro and indicators of oxidative stress (MDA, H_2_O_2_, and EL) were also observed. However, in the *P. fungorum* and *Delftia* sp. inoculated plants, the growth, biomass production, and RWC were increased; in contrast, they decremented the Pro, MDA, H_2_O_2_, and EL levels. The retardation of the oxidative stress indicators correlated with the enhanced activities of antioxidant enzymes, such as APX, MDHAR, DHAR, GR, GPX, and CAT in the bacteria-inoculated plants under Cd toxicity. Another effect of Cd is the accelerated production of MG, which is also mitigated by the augmentation of Gly I and Gly II activities in the Cd-stressed plants. Moreover, it is visible from the current study that the probiotic bacteria *P. fungorum* and *Delftia* sp. have the potential to alleviate the Cd stress through modulating ROS scavenging, antioxidant defense system, and glyoxalase system. Therefore, further experimentation should be conducted to explore the molecular mechanisms of the *P. fungorum* BRRh-4 and *Delftia* sp. BTL-M2 on the Cd tolerance and the practical application on the contaminated sites to remediate the Cd contamination in an eco-friendly way.

## 5. Conclusions

The current study demonstrated the amelioration of the stressful condition of Cd on rapeseed plants’ growth, physiology, and biochemical properties through the inoculation of two plant growth-promoting probiotic bacteria, *P. fungorum* BRRh-4 and *Delftia* sp. BTL-M2. Under Cd stress, the rapeseed plants showed increased accumulation of ROS, lipid peroxidation, disrupted glyoxalase system, and impaired physiological activities. Upon inoculation with the bacteria in the presence of Cd augmented the antioxidant defense and glyoxalase enzymes, thus mitigating the pernicious effect of ROS and reducing lipid peroxidation in the Cd-stressed plants. Moreover, prevention of pigment damage and improved water status enhanced growth, and biomass accumulation in the bacterial inoculated Cd-stressed plants, which ultimately affected the incrimination of the yield components of rapeseed.

## Data Availability

All data are available in this article.

## References

[1] Palansooriya K.N., Shaheen S.M., Chen S.S., Tsang D.C., Hashimoto Y., Hou D., Bolan N.S., Rinklebe J., Ok Y.S. (2020). Soil amendments for immobilization of potentially toxic elements in contaminated soils: A critical review. Environ. Int..

[2] Gill S.S., Anjum N.A., Hasanuzzaman M., Gill R., Trived D.K., Ahmad I., Pereira E., Tuteja N. (2013). Glutathione reductase and glutathione: A boon in disguise for plant abiotic stress defense operations. Plant Physiol. Biochem..

[3] Silambarasan S., Logeswari P., Vangnai A.S., Kamaraj B., Cornejo P. (2022). Plant growth-promoting actinobacterial inoculant assisted phytoremediation increases cadmium uptake in *Sorghum bicolor* under drought and heat stresses. Environ. Pollut..

[4] Hasanuzzaman M., Parvin K., Bardhan K., Nahar K., Anee T.I., Masud A.A.C., Fotopoulos V. (2021). Biostimulants for the regulation of reactive oxygen species metabolism in plants under abiotic stress. Cells.

[5] Hasanuzzaman M., Bhuyan M.H.M.B., Zulfiqar F., Raza A., Mohsin S.M., Mahmud J.A., Fujita M., Fotopoulos V. (2020). Reactive oxygen species and antioxidant defense in plants under abiotic stress: Revisiting the crucial role of a universal defense regulator. Antioxidants.

[6] Khan E.A., Ahmed H.M.I., Misra M., Sharma P., Misra A.N., Hasanuzzaman M. (2022). Nitric oxide alleviates cadmium-impeded growth by limiting ROS accumulation in pea seedlings. BIOCELL.

[7] Clemens S., Ma J.F. (2016). Toxic heavy metal and metalloid accumulation in crop plants and foods. Annu. Rev. Plant Biol..

[8] Ghori N.H., Ghori T., Hayat M.Q., Imadi S.R., Gul A., Altay V., Ozturk M. (2019). Heavy metal stress and responses in plants. Int. J. Environ. Sci. Technol..

[9] Hasanuzzaman M., Bhuyan M.H.M.B., Anee T.I., Parvin K., Nahar K., Mahmud J.A., Fujita M. (2019). Regulation of ascorbate-glutathione pathway in mitigating oxidative damage in plants under abiotic stress. Antioxidants.

[10] Chen Y., Chao Y., Li Y., Lin Q., Bai J., Tang L., Wang S., Ying R., Qiu R. (2016). Survival strategies of the plant-associated bacterium *Enterobacter* sp. strain EG16 under cadmium stress. Appl. Environ. Microbiol..

[11] Sharma P., Chouhan R., Bakshi P., Gandhi S.G., Kaur R., Sharma A., Bhardwaj R. (2022). Amelioration of chromium-induced oxidative stress by combined treatment of selected plant-growth-promoting rhizobacteria and earthworms via modulating the expression of genes related to reactive oxygen species metabolism in *Brassica juncea*. Front. Microbiol..

[12] Khoshru B., Mitra D., Khoshmanzar E., Myo E.M., Uniyal N., Mahakur B., Mohapatra P.K.D., Panneerselvam P., Boutaj H., Alizadeh M. (2020). Current scenario and future prospects of plant growth-promoting rhizobacteria: An economic valuable resource for the agriculture revival under stressful conditions. J. Plant Nutr..

[13] Sahile A.A., Khan M.A., Hamayun M., Imran M., Kang S.-M., Lee I.-J. (2021). Novel *Bacillus cereus* strain, ALT1, enhance growth and strengthens the antioxidant system of soybean under cadmium stress. Agronomy.

[14] Chi Y., You Y., Wang J., Chen X., Chu S., Wang R., Zhang X., Yin S., Zhang D., Zhou P. (2022). Two plant growth-promoting bacterial *Bacillus* strains possess different mechanisms in affecting cadmium uptake and detoxification of *Solanum nigrum* L. Chemosphere.

[15] Saeed Z., Naveed M., Imran M., Bashir M.A., Sattar A., Mustafa A., Hussain A., Xu M. (2019). Combined use of *Enterobacter* sp. MN17 and zeolite reverts the adverse effects of cadmium on growth, physiology and antioxidant activity of *Brassica napus*. PLoS ONE.

[16] Tanwir K., Javed M.T., Abbas S., Shahid M., Akram M.S., Chaudhary H.J., Iqbal M. (2021). *Serratia* sp. CP-13 alleviates Cd toxicity by morpho-physio-biochemical improvements, antioxidative potential and diminished Cd uptake in *Zea mays* L. cultivars differing in Cd tolerance. Ecotoxicol. Environ. Saf..

[17] Sepehri M., Khatabi B. (2021). Combination of siderophore-producing bacteria and *Piriformospora indica* provides an efficient approach to improve cadmium tolerance in alfalfa. Microbial. Ecol..

[18] Mourato M.P., Moreira I.N., Leitão I., Pinto F.R., Sales J.R., Martins L.L. (2015). Effect of heavy metals in plants of the genus *Brassica*. Int. J. Mol. Sci..

[19] Ahmad P., Sarwat M., Bhat N.A., Wani M.R., Kazi A.G., Tran L.P. (2015). Alleviation of cadmium toxicity in *Brassica juncea* L. (Czern. & Coss.) by calcium application involves various physiological and biochemical strategies. PLoS ONE.

[20] Saghafi D., Ghorbanpour M., Ajiloo H.S., Lajayer B.A. (2019). Enhancement of growth and salt tolerance in *Brassica napus* L. seedlings by halotolerant *Rhizobium* strains containing ACC-deaminase activity. Ind. J. Plant Physiol..

[21] Stassinos P.M., Rossi M., Borromeo I., Capo C., Beninati S., Forni C. (2022). Amelioration of salt stress tolerance in rapeseed (*Brassica napus*) cultivars by seed inoculation with *Arthrobacter globiformis*. Plant Biosyst..

[22] Braña V., Cagide C., Morel M.A., Castro-Sowinski S. (2016). The sustainable use of *Delftia* in agriculture, bioremediation, and bioproducts synthesis. Microbial Models: From Environmental to Industrial Sustainability.

[23] Khan M.M.A., Haque E., Paul N.C., Khaleque M.A., Al-Garni S.M., Rahman M., Islam M.T. (2017). Enhancement of growth and grain yield of rice in nutrient deficient soils by rice probiotic bacteria. Rice Sci..

[24] Rahman M., Sabir A.A., Mukta J.A., Khan M., Alam M., Mohi-Ud-Din M., Miah M., Rahman M., Islam M.T. (2018). Plant probiotic bacteria *Bacillus* and *Paraburkholderia* improve growth, yield and content of antioxidants in strawberry fruit. Sci. Rep..

[25] Liu X.X., Hu X., Cao Y., Pang W.J., Huang J.Y., Guo P., Huang L. (2019). Biodegradation of phenanthrene and heavy metal removal by acid-tolerant *Burkholderia fungorum* FM-2. Front. Microbiol..

[26] Morel M.A., Ubalde M.C., Braña V., Castro-Sowinski S. (2011). *Delftia* sp. JD2: A potential Cr(VI)-reducing agent with plant growth-promoting activity. Arch. Microbiol..

[27] BARI (Bangladesh Agricultural Research Institute) (2021). Krishi Projukti Hatboi.

[28] Barrs H.D., Weatherley P.E. (1962). A re-examination of the relative turgidity technique for estimating water deficits in leaves. Aust. J. Biol. Sci..

[29] Bates L.S., Waldren R.P., Teari D. (1973). Rapid determination of free proline for water stress studies. Plant Soil.

[30] Dionisio-Sese M.L., Tobita S. (1998). Antioxidant responses of rice seedlings to salinity stress. Plant Sci..

[31] Heath R.L., Packer L. (1968). Photoperoxidation in isolated chloroplast. I. Kinetics and stoichiometry of fatty acid peroxidation. Arch. Biochem. Biophys..

[32] Yu C.W., Murphy T.M., Lin C.H. (2003). Hydrogen peroxide induced chilling tolerance in mung beans mediated through ABA-independent glutathione accumulation. Funct. Plant Biol..

[33] Hasanuzzaman M., Raihan M.R.H., Khojah E., Samra B.N., Fujita M., Nahar K. (2021). Biochar and chitosan regulate antioxidant defense and methylglyoxal detoxification systems and enhance salt tolerance in jute (*Corchorus olitorius* L.). Antioxidants.

[34] Bradford M.M. (1976). A rapid and sensitive method for the quantitation of microgram quantities of protein utilizing the principle of protein-dye binding. Anal. Biochem..

[35] Nakano Y., Asada K. (1981). Hydrogen peroxide is scavenged by ascorbate-specific peroxidase in spinach chloroplasts. Plant Cell Physiol..

[36] Hossain M.A., Nakano Y., Asada K. (1984). Monodehydroascorbate reductase in spinach chloroplasts and its participation in the regeneration of ascorbate for scavenging hydrogen peroxide. Plant Cell Physiol..

[37] Elia A.C., Galarini R., Taticchi M.I., Dorr A.J.M., Mantilacci L. (2003). Antioxidant responses and bioaccumulation in *Ictalurus melas* under mercury exposure. Ecotoxicol. Environ. Saf..

[38] Principato G.B., Rosi G., Talesa V., Govannini E., Uolila L. (1987). Purification and characterization of two forms of glyoxalase II from rat liver and brain of Wistar rats. Biochim. Biophys. Acta.

[39] (2008). CoStat—Statistics Software.

[40] Kumar M., Giri V.P., Pandey S., Gupta A., Patel M.K., Bajpai A.B., Jenkins S., Siddique K.H.M. (2021). Plant-growth-promoting rhizobacteria emerging as an effective bioinoculant to improve the growth, production, and stress tolerance of vegetable crops. Int. J. Mol. Sci..

[41] Gupta A., Rai S., Bano A., Khanam A., Sharma S., Pathak N. (2021). Comparative evaluation of different salt-tolerant plant growth promoting bacterial isolates in mitigating the induced adverse effect of salinity in *Pisum sativum*. Biointerface Res. Appl. Chem..

[42] Xu Q., Pan W., Zhang R., Lu Q., Xue W., Wu C., Song B., Du S. (2018). Inoculation with *Bacillus subtilis* and *Azospirillum brasilense* produces abscisic acid that reduces Irt1-mediated cadmium uptake of roots. J. Agric. Food Chem..

[43] Li Y., Mo L., Zhou X., Yao Y., Ma J., Liu K., Yu F. (2021). Characterization of plant growth-promoting traits of *Enterobacter* sp. and its ability to promote cadmium/lead accumulation in *Centella asiatica* L. Environ. Sci. Pollut. Res..

[44] Gill S.S., Tuteja N. (2010). Reactive oxygen species and antioxidant machinery in abiotic stress tolerance in crop plants. Plant Physiol. Biochem..

[45] Naveed M., Ramzan N., Mustafa A., Samad A., Niamat B., Yaseen M., Ahmad Z., Hasanuzzaman M., Sun N., Shi W. (2020). Alleviation of salinity induced oxidative stress in *Chenopodium quinoa* by Fe biofortification and biochar—Endophyte interaction. Agronomy.

[46] Chen M., Yang Z., Liu J., Zhu T., Wang B. (2018). Adaptation mechanism of salt excluders under saline conditions and its applications. Int. J. Mol. Sci..

[47] Jan R., Khan M.A., Asaf S., Lee I.J., Kim K.M. (2019). Metal resistant endophytic bacteria reduces cadmium, nickel toxicity, and enhances expression of metal stress related genes with improved growth of *Oryza sativa*, via regulating its antioxidant machinery and endogenous hormones. Plants.

[48] Bhuyan M.H.M.B., Parvin K., Mohsin S.M., Mahmud J.A., Hasanuzzaman M., Fujita M. (2020). Modulation of cadmium tolerance in rice: Insight into vanillic acid-induced upregulation of antioxidant defense and glyoxalase systems. Plants.

[49] Lastochkina O., Aliniaeifard S., Garshina D., Garipova S., Pusenkova L., Allagulova C., Fedorova K., Baymiev A., Koryakov I., Sobhani M. (2021). Seed priming with endophytic *Bacillus subtilis* strain-specifically improves growth of *Phaseolus vulgaris* plants under normal and salinity conditions and exerts anti-stress effect through induced lignin deposition in roots and decreased oxidative and osmotic damages. J. Plant Physiol..

[50] Ji C., Tian H., Wang X., Song X., Ju R., Li H., Gao Q., Li C., Zhang P., Li J. (2020). *Bacillus subtilis* HG-15, a halotolerant rhizoplane bacterium, promotes growth and salinity tolerance in wheat (*Triticum aestivum*). BioMed Res. Int..

[51] Rashid M.I., Mujawar L.H., Shahzad T., Almeelbi T., Ismail I.M.I., Oves M. (2016). Bacteria and fungi can contribute to nutrients bioavailability and aggregate formation in degraded soils. Microbiol. Res..

[52] Ha-Tran D.M., Nguyen T.T.M., Hung S.-H., Huang E., Huang C.-C. (2021). Roles of Plant growth-promoting rhizobacteria (PGPR) in stimulating salinity stress defense in plants: A Review. Int. J. Mol. Sci..

[53] Ullah S., Kolo Z., Egbichi I., Keyster M., Ludidi N. (2016). Nitric oxide influences glycine betaine content and ascorbate peroxidase activity in maize. S. Afr. J. Bot..

[54] Zeng X., Pang L., Chen Y., Kong X., Chen J., Tian X. (2020). Bacteria *Sphingobium yanoikuyae* Sy310 enhances accumulation capacity and tolerance of cadmium in *Salix matsudana* Koidz roots. Environ. Sci. Pollut. Res..

[55] Ullah I., Al-Johny B.O., Al-Ghamdi K.M., Al-Zahrani H.A., Anwar Y., Firoz A., Naser A.K., Almatry M.A.A. (2019). Endophytic bacteria isolated from *Solanum nigrum* L., alleviate cadmium (Cd) stress response by their antioxidant potentials, including SOD synthesis by *sodA* gene. Ecotoxicol. Environ. Saf..

[56] Puthiyottil P., Akkara Y. (2021). Pre-treatment with *Bacillus subtilis* mitigates drought induced photo-oxidative damages in okra by modulating antioxidant system and photochemical activity. Physiol. Mol. Biol. Plants.

[57] Das B.K., Kumar A., Maindola P., Mahanty S., Jain S.K., Reddy M.K., Arockiasamy A. (2016). Non-native ligands define the active site of *Pennisetum glaucum* (L.) R. Br dehydroascorbate reductase. Biochem. Biophys. Res. Commun..

[58] Abd_Allah E.F., Alqarawi A.A., Hashem A., Radhakrishnan R., Al-Huqail A.A., Al-Otibi F.O.N., Malik J.A., Alharbi R.I., Egamberdieva D. (2018). Endophytic bacterium *Bacillus subtilis* (BERA 71) improves salt tolerance in chickpea plants by regulating the plant defense mechanisms. J. Plant Interact..

[59] Khan A.L., Bilal S., Halo B.A., Al-Harrasi A., Khan A.R., Waqas M., Al-Thani G.S., Al-Amri I., Al-Rawahi A., Lee I.-J. (2017). *Bacillus amyloliquefaciens* BSL16 improves phytoremediation potential of *Solanum lycopersicum* during copper stress. J. Plant Interact..

[60] Rahman M., Rahman K., Sathi K.S., Alam M.M., Nahar K., Fujita M., Hasanuzzaman M. (2021). Supplemental selenium and boron mitigate salt-induced oxidative damages in *Glycine max* L. Plants.

[61] Siddiqui M.H., Alamri S., Alsubaie Q.D., Ali H.M. (2020). Melatonin and gibberellic acid promote growth and chlorophyll biosynthesis by regulating antioxidant and methylglyoxal detoxification system in tomato seedlings under salinity. J. Plant Growth Regul..

[62] Hernández J.A., Barba-Espín G., Diaz-Vivancos P., Hossain M., Mostofa M., Diaz-Vivancos P., Burritt D., Fujita M., Tran L.S. (2017). Glutathione-mediated biotic stress tolerance in plants. Glutathione in Plant Growth, Development, and Stress Tolerance.

[63] Naveed M., Mustafa A., Majeed S., Naseem Z., Saeed Q., Khan A., Nawaz A., Baig K.S., Chen J.T. (2020). Enhancing cadmium tolerance and pea plant health through *Enterobacter* sp. MN17 inoculation together with biochar and gravel sand. Plants.

[64] Kumar A., Singh S., Mukherjee A., Rastogi R.P., Verma J.P. (2021). Salt-tolerant plant growth-promoting *Bacillus pumilus* strain JPVS11 to enhance plant growth attributes of rice and improve soil health under salinity stress. Microbiol. Res..

[65] Tian Z., Li J., Jia X., Yang F., Wang Z. (2016). Assimilation and translocation of dry matter and phosphorus in rice genotypes affected by salt-alkaline stress. Sustainability.

[66] Zhou X., Liu X., Zhao J., Guan F., Yao D., Wu N., Tian J. (2021). The endophytic bacterium *Bacillus koreensis* 181–22 promotes rice growth and alleviates cadmium stress under cadmium exposure. Appl. Microbiol. Biotechnol..

